# Error Correction of the RapidEye Sub-Pixel Correlation: A Case Study of the 2019 Ridgecrest Earthquake Sequence

**DOI:** 10.3390/s24144726

**Published:** 2024-07-21

**Authors:** Wulinhong Luo, Qi An, Guangcai Feng, Zhiqiang Xiong, Lijia He, Yilin Wang, Hongbo Jiang, Xiuhua Wang, Ning Li, Wenxin Wang

**Affiliations:** School of Geosciences and Info-Physics, Central South University, Changsha 410083, China; luowulinhong@csu.edu.cn (W.L.); anqi27@csu.edu.cn (Q.A.); zqxiong_flhs@csu.edu.cn (Z.X.); lijiahe@csu.edu.cn (L.H.); 235011017@csu.edu.cn (Y.W.); cianghungbo@csu.edu.cn (H.J.); csuxhgd@csu.edu.cn (X.W.); ning_li@csu.edu.cn (N.L.); wangwx@csu.edu.cn (W.W.)

**Keywords:** RapidEye, sub-pixel correlation, error analysis, Ridgecrest earthquake sequence

## Abstract

The optical image sub-pixel correlation (SPC) technique is an important method for monitoring large-scale surface deformation. RapidEye images, distinguished by their short revisit period and high spatial resolution, are crucial data sources for monitoring surface deformation. However, few studies have comprehensively analyzed the error sources and correction methods of the deformation field obtained from RapidEye images. We used RapidEye images without surface deformation to analyze potential errors in the offset fields. We found that the errors in RapidEye offset fields primarily consist of decorrelation noise, orbit error, and attitude jitter distortions. To mitigate decorrelation noise, the careful selection of offset pairs coupled with spatial filtering is essential. Orbit error can be effectively mitigated by the polynomial fitting method. To address attitude jitter distortions, we introduced a linear fitting approach that incorporated the coherence of attitude jitter. To demonstrate the performance of the proposed methods, we utilized RapidEye images to extract the coseismic displacement field of the 2019 Ridgecrest earthquake sequence. The two-dimensional (2D) offset field contained deformation signals extracted from two earthquakes, with a maximum offset of 2.8 m in the E-W direction and 2.4 m in the N-S direction. A comparison with GNSS observations indicates that, after error correction, the mean relative precision of the offset field improved by 92% in the E-W direction and by 89% in the N-S direction. This robust enhancement underscores the effectiveness of the proposed error correction methods for RapidEye data. This study sheds light on large-scale surface deformation monitoring using RapidEye images.

## 1. Introduction

Sub-pixel correlation (SPC) is an important technique for surface deformation monitoring. It calculates the pixel offsets across two optical images [1]. While initially used for the acquisition of glacier flow velocity fields [2], this technique gradually became used for the monitoring of other surface deformations. As optical satellite data availability and diversity have expanded, SPC has evolved from obtaining deformation information based on a single image pair to analyzing the surface deformation time series using multi-images [3,4,5]. Nowadays, the SPC technique has been widely used in the monitoring of dune migration [6,7], earthquakes [8,9,10,11], glacial movement [12,13], landslides [14,15,16], and so on. However, the offset fields obtained by SPC have many error sources, such as spatial-temporal decorrelated noise, long wavelength orbit error, satellite attitude jitter distortions, stripe artifacts, and topographic shadow artifacts [17,18]. These errors limit the deformation monitoring accuracy, subsequently affecting deformation signal extraction and characterization. Therefore, studying the causes and correction methods of these errors in the SPC-derived offset fields is of great significance for the application and development of optical images.

When addressing the removal of stripe errors in single remote sensing images, researchers typically rely on grayscale analysis to correct these errors. These studies often employ edge-detection and line-tracing techniques to identify and eliminate stripe errors [19,20,21]. These methods are highly automated, rapid, and efficient. However, their applicability in the offset field is limited. In surface deformation monitoring, it is crucial to consider the deformation characteristics of the study area. For instance, manually masking deformation regions is necessary to prevent the loss of deformation information. Errors in the offset fields acquired by SPC are usually related to the satellite imaging principle, range, and conditions. It is essential to investigate the systematic error composition and correction methods for different satellites. SPOT imagery’s stripe error in the offset field can be effectively removed using the mean subtraction method [22]. The stripe artifacts of Landsat 8, produced during the push-broom imaging process, can be removed by limiting the spatial baseline and radiant energy [17]. PlanetScope imagery, which faces discontinuities in deformation values between frames due to frame imaging, can effectively resolve this issue using an approach based on polynomial surface fitting [23]. Considering the imaging principle of Sentinel-2, the optimized “Mean elimination method” can effectively mitigate satellite attitude jitter distortions [18]. Therefore, the error sources and correction methods differ among optical images acquired by various satellites.

RapidEye satellites were successfully launched by Germany in August 2008 and were in orbit for 11 years until March 2020, accumulating a large amount of archived data for surface deformation monitoring. Boasting high temporary and spatial resolutions, these images offer multi-spectral information [24,25] and hold promising applications in earth observations. Based on abundant spectral information, RapidEye data have been widely used in crop growth monitoring [26,27], feature identification and classification [28,29], and feature information extraction [30,31]. In geo-disaster research, RapidEye imagery, owing to its high temporal and spatial resolution, is widely used for landslide identification and inventory [32,33,34,35,36]. Furthermore, utilizing the SPC technique, RapidEye imagery can extract large-scale surface deformation information, making it an essential data source for the quantitative analysis of geo-disasters, such as earthquakes, glacier movements, and large-scale landslides. However, systematic errors within displacement fields significantly constrain the accuracy of deformation monitoring, yet there remains a lack of dedicated research to address this issue. To date, RapidEye has predominantly been applied to small-scale landslide deformation analysis [14], which postulated that the stacking of large sets of offset maps should compensate for this topographic bias. Errors within small-scale offset fields are typically singular and easily corrected; thus, very few systematic discussions have been conducted on them. Conversely, large-scale offset fields are characterized by complex and severe systematic errors. In conclusion, developing systematic error correction methods for large-scale offset fields derived from RapidEye is crucial for conducting quantitative surface deformation monitoring studies using RapidEye data. Significant disparities exist in error correction methods for offset fields derived from various satellites, underscoring the critical necessity for a holistic error correction framework tailored to RapidEye. Such a framework is designed to enhance the precision and efficiency of geo-disaster deformation studies utilizing RapidEye imagery, thereby maximizing the application potential of RapidEye data in geo-disaster management and monitoring.

To address the aforementioned issues, we selected the Ridgecrest area in California, USA, as our study area to analyze the error characteristics of the offset fields obtained from RapidEye images and propose corresponding correction methods applied to the earthquakes that occurred in July 2019 in Ridgecrest. Additionally, the surface deformation monitoring capability of RapidEye is also discussed.

## 2. Study Area

**Figure 1:**
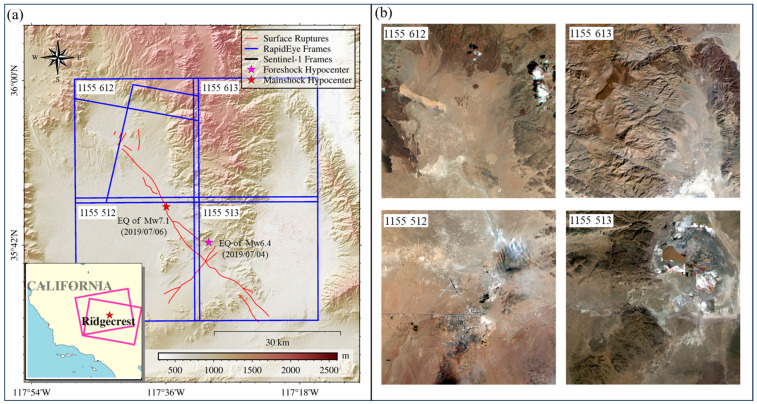
Study area and corresponding RapidEye images. (**a**) Study area and image coverage. The blue rectangles show the coverage of the RapidEye images. The stars are the epicenters of the 2019 Ridgecrest earthquake from the USGS. The magenta rectangles are Sentinel-1 coverage. The red lines indicate the active faults. (**b**) True-color RapidEye images over the study area.

Ridgecrest is located in Kern County in California, United States. The region experienced an earthquake sequence in 2019, making it ideal for capturing surface deformation information using SPC effectively. The earthquake sequence included an Mw 6.4 foreshock on 4 July 2019 at 17:33 UTC and an Mw 7.1 mainshock that occurred 34 h later. The Mw 6.4 earthquake, dominated by left-lateral strike-slip motions, ruptured, reaching a length of 10 km. The Mw 7.1 earthquake, dominated by right-lateral strike-slip motions, ruptured, reaching a length of 49 km. RapidEye images in this area are sufficient for thorough analysis. Additionally, GNSS stations in this area can verify the error correction method proposed in this study. Therefore, this region is suitable for investigating RapidEye imagery error sources, proposing correction methods, and assessing the effectiveness of surface deformation monitoring after correction. The RapidEye image coverage of the study area is shown in Figure 1.

## 3. Data and Methods

### 3.1. Data

The RapidEye constellation-based optical remote sensing satellite system possesses unique capabilities, consisting of five satellites with identical parameter configurations. These satellites operate in a sun-synchronous orbit, enabling the imaging of any area on Earth within a single day, with a revisit period as short as one day. Each satellite is equipped with a five-band multi-spectral optical imager featuring a swath width of 80 km and a spatial resolution of up to 5 m. The RapidEye satellites accumulated a substantial archive of images over nearly 11 years of operation. Satellite parameters are detailed in Table 1.

### 3.2. Methods

The methodology comprises four parts. First, the optimal experimental bands are determined. Second, potential errors in the offset field are analyzed. Third, corresponding correction methods for each error component are proposed. Finally, based on the content of the first three parts, a comprehensive workflow for surface deformation monitoring using RapidEye is developed.

#### 3.2.1. The Optimal Band of RapidEye Images for Surface Offset Monitoring

The band radiation characteristics determine the effect of surface deformation monitoring. In this study, images acquired in the period without deformation (Table 2) were used to identify the optimal band for monitoring surface deformation. Typically, the offset in the initial offset field is the sum of decorrelation noise, attitude jitter distortions, and terrain shadow, among others. The error levels of offset fields acquired from different bands reflect the robustness of surface deformation monitoring. Here, the root-mean-square error (RMSE) of the deformation values serves as an accuracy evaluation index, guiding the selection of the optimal band for deformation monitoring.

The RMSE of each offset field (Table 3) was obtained from the images in Table 2 by SPC with COSI-Corr 1.5 software. Band 5 has the highest percentage of minimum RMSE in the east–west (E-W) and north–south (N-S) offset fields and the minimum mean RMSE in all the offset fields. Therefore, Band 5 was selected as the optimal band for error analysis and deformation monitoring.

#### 3.2.2. Errors Analysis

In space, satellite imaging faces challenges such as space debris, solar wind, and various types of radiation, which can change satellite flight orbits and imaging attitudes, eventually leading to errors in the offset field obtained by SPC. These errors are usually not fully presented in a single offset field, so this study examined four RapidEye image pairs acquired before and one pair after the earthquake to analyze the error of the initial offset fields in the E-W direction, the N-S direction, and the signal-to-noise ratio (SNR) obtained by COSI-Corr.

**Figure 2:**
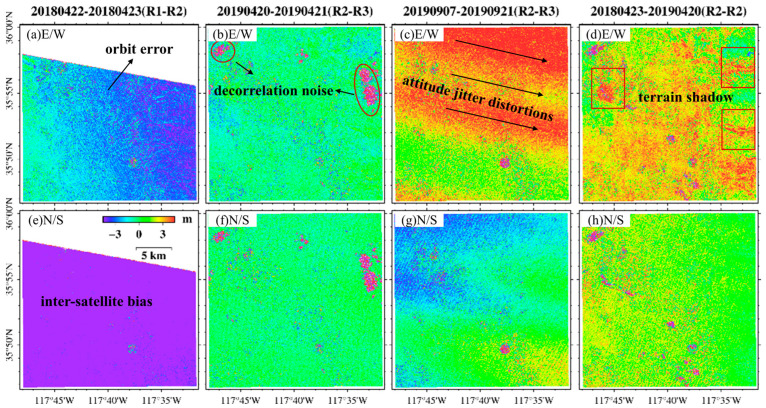
Error components of RapidEye images in the E-W and N-S offset fields. R1, R2, and R3 represent satellite numbers. The error with a systematic trend in (**a**) is an orbital error. The collective deviation depicted in (**e**) is attributed to inter-satellite bias. The outlier in (**b**,**f**) is decorrelation noise. The trend errors perpendicular to the flight direction in (**c**,**g**) are attitude jitter distortions. The errors enclosed within the red rectangular (**d**) are terrain shadows. Figure (**h**) includes decorrelation noise and terrain shadow.

RapidEye images containing fewer clouds before the event were selected for imaging groups (Table 4). COSI-Corr was utilized to acquire the offset field of these image pairs. The parameters of COSI-Corr software for all experiments and for the cross-correlation calculation are as follows: in the frequency domain, the initial sliding window is 64 × 64 pixels, the final sliding window is 32 × 32 pixels, and the sliding step size is 2 pixels (10 m). The iteration repeats two times, and the mask threshold is 0.9. See Figure 2.

The offset fields obtained by SPC mainly contain decorrelated noise, orbital error, attitude jitter distortions, inter-satellite bias, and terrain shadow disturbances.

#### 3.2.3. Correction Methods

Decorrelation noise primarily arises from the changes in light conditions and surface radiation intensity. Environmental factors, such as radiation intensity, cloud cover, rain, snow, and other natural conditions, induce changes in surface radiation intensity. These changes lead to the absence of image texture features, resulting in decorrelation noise. Image texture features are an important basis for extracting surface information from optical images. Inaccurate texture information can reduce the accuracy of the final offset field.

Two common methods for removing decorrelated noise are as follows: (1) Setting the SNR threshold to exclude the region with low SNR. Usually, the regions with an SNR lower than 0.9 are masked. (2) Wide regions affected by decorrelation can also be eliminated, such as those caused by clouds, waters, and vegetation.

**Figure 3:**
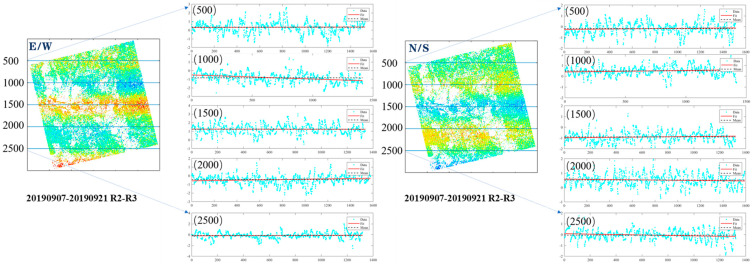
The characteristic of attitude jitter distortions of RapidEye. The blue discrete points are the data along the profile line, the black dashed line is the mean value of these data, and the red solid line is the fitted straight line considering the asymptotic tendency of the attitude jitter stripes. R2 and R3 denote the satellite numbers.

An orbital error is a trending systematic bias that is affected by the accuracy and stability of the satellite orbit. Preliminary ortho-correction and geometric correction can weaken the effect of this error, but they are insufficient to entirely overcome the systematic bias in images acquired at different times. Notably, there is also a clear trending orbital error in the offset results obtained from RapidEye images (Figure 3), which can be removed using the polynomial fitting method [37,38].

*D* = *a*_0_ + *a*_1_ *x* + *a*_2_ *y* (1)

where *D* is the fitted orbit error, *a*_0_, *a*_1_, and *a*_2_ are the unknown parameters, and *x*, *y* are the row and column numbers or coordinates of the deformation matrix.

Attitude jitter distortion is a periodic error streak caused by attitude jitter along the satellite’s vertical flight direction. It is caused by external forces in space, such as solar activity and star gravity, coupled with the down-sampling of satellite imaging attitudes. The traditional method of “Mean value elimination” [22,39] can eliminate attitude jitter distortion in a convenient and efficient way. However, its applicability varies depending on the imaging principle of various satellites. By analyzing the error source of RapidEye, we found that its attitude jitter distortions have a gradual trend along the vertical flight direction, and the traditional mean value elimination method is not applicable. This type of asymptotic tendency is particularly prominent in the offset field. Figure 3 illustrates the gradual trend observed in attitude jitter distortions along the vertical flight direction of the offset matrix.

Based on the above characteristics, we considered consistency along the parallel flight direction when processing n rows offset by the matrix, as shown below:

*Y_i_* = *P*(1)*_i_ X_i_* + *P*(2)*_i_* (*i* = 1, …, n) (2)

Δ*y* = |*Y_i_*_+1_| − |*Y_i_*| (3)

**Figure 4:**
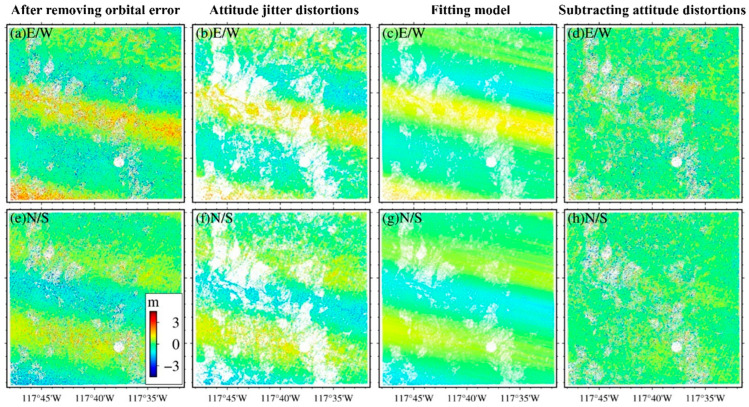
The correction of attitude jitter distortions of RapidEye. (**a**,**e**) are the offset fields with attitude jitter distortions. (**b**,**f**) are the offset fields after filtering. (**c**,**g**) present the attitude jitter distortions models based on the filtered offset fields computed by Equations (2)–(4). (**d**,**h**) are the offset fields after removing attitude jitter distortions.

*Y_i_* = *Y_i+_*_1_ − *sign* × *E*(Δ*y*) (4)

where *P*(1), *P*(2) are unknown parameters, $Y_{i}$ is the fitting matrix used to eliminate the attitude jitter distortions, Δ*y* ensures the coherence of attitude jitter along the parallel flight direction, and sign is the positive and negative sign matrices in the same dimension as the offset matrix. The error correction effect is shown in Figure 4.

Inter-satellite bias is a systematic error introduced by different imaging conditions among satellites of the RapidEye constellation during earth observation. When the error magnitude is small, it can be removed by the improved “mean elimination method”. When the error magnitude is large enough to completely mask the surface deformation information, it can be rejected directly.

#### 3.2.4. The Processing Workflow in Practical Applications

**Figure 5:**
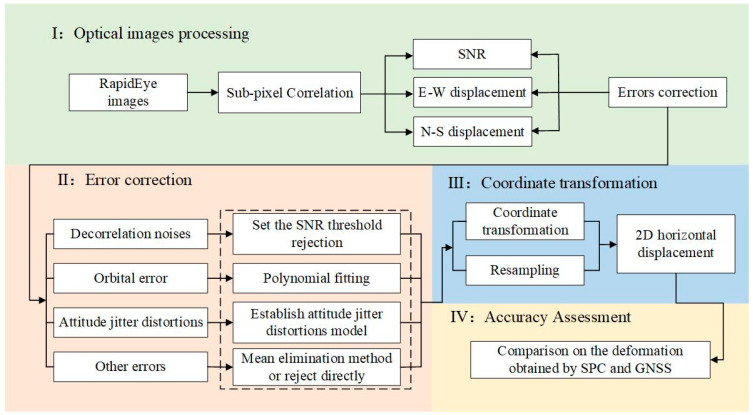
Processing flow chart of RapidEye images.

Based on the error correction method proposed above, we developed a comprehensive processing workflow to obtain surface deformation information from RapidEye imagery SPC, as shown in Figure 5. Firstly, the initial E-W displacement, initial N-S displacement, and SNR were obtained. Then, error correction was performed. The corrected offset results underwent a coordinate transformation and resampling to generate the two-dimensional (2D) horizontal offset field of the study area. Finally, an accuracy assessment was conducted by comparing GNSS observations in the study area with the offset values. RapidEye data information on the 2019 Ridgecrest earthquake is shown in Table 5. The parameters for COSI-corr are identical to those previously mentioned.

## 4. Result

**Figure 6:**
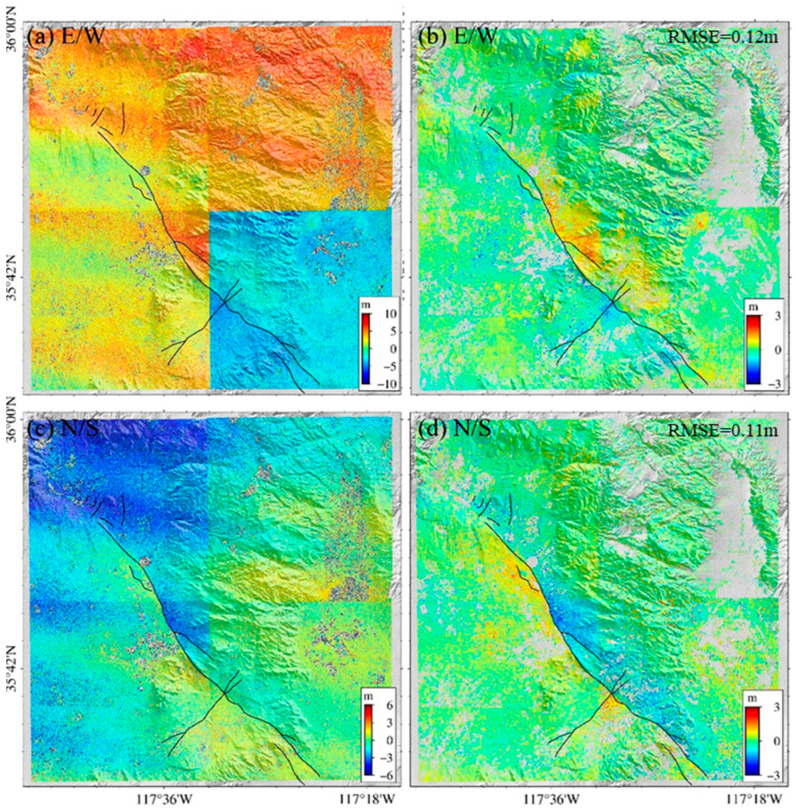
E-W and N-S components of the offset field before and after error correction. (**a**,**c**) show the offset fields before error correction. (**b**,**d**) show the offset fields after error correction.

Following the workflow outlined in Figure 5, we obtained the offset field of the study area. Optical displacement fields before and after error elimination are displayed in Figure 6. Due to different imaging periods and conditions, the raw offset fields had serious orbital errors (Figure 6a,c). As Figure 6 shows, feature radiance intensity exhibits a clear difference, leading to a significant lack of consistency. Trend inaccuracies resulting from satellite attitude instability are significant. These errors directly mask a large portion of information related to surface deformation. After error correction, decorrelation noise, orbital errors, and satellite attitude jitter distortions are effectively eliminated, facilitating the retrieval of deformation signals (Figure 6b,d). The 2D offset field contains the deformation signals extracted from two earthquakes, featuring an average displacement of 0.67 m and a maximum displacement of 2.8 m in the E-W direction as well as an average displacement of 0.71 m and a maximum displacement of 2.4 m. The predominant slip was on the NE-SW fault. Notably, in the vicinity of the main shock’s epicenter, N-S displacement surpassed the E-W displacement. A right-lateral strike-slip motion during the main shock was indicated by the E-W deformation, which manifested as westward displacement on the main shock’s south side and overall eastward displacement to the north. The Mw7.4 earthquake, the main shock, was dominated by the right-lateral strike-slip. Our findings align well with those of previous studies [40,41], showing good concordance in fault trace details and deformation magnitude.

**Figure 7:**
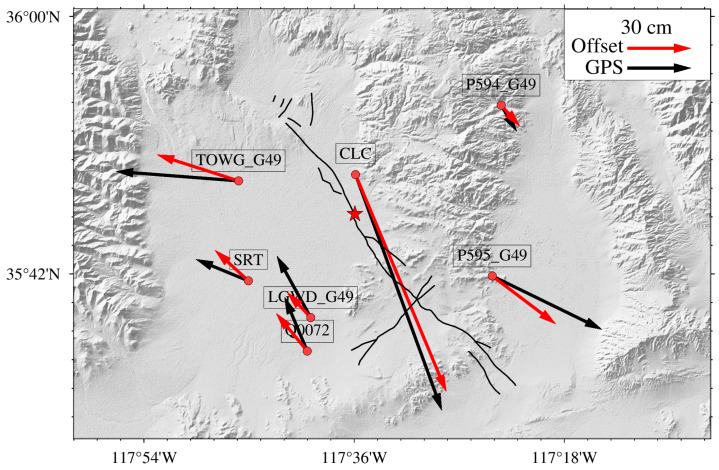
Comparison of the deformation value of Mw 7.1 obtained by SPC and GNSS. (Red dots represent GNSS station locations, purple pentagrams represent the foreshock epicenter, the red star represent the main shock epicenter, black arrows represents the GNSS observations, and red arrows represent the observations calculated from RapidEye imagery).

In the study area, numerous GNSS stations are deployed on both sides of the ruptured fault (https://www.unavco.org/highlights/2019/ridgecrest.html, accessed on 30 August 2019), which can be used to assess the accuracy of the offset field. GNSS monitoring data separate the deformation signals from the two earthquakes; for example, the GNSS monitoring data in Figure 7 exclusively captured signals from the main shock. However, the offset field obtained from RapidEye contains signals of deformation caused by both earthquakes, such as the time for the master and slave images to cover both earthquakes. Therefore, here, we only compare the GNSS monitoring data following the main shock occurrence with the 2D offset field obtained from RapidEye images. We calculated the standard deviations of the E-W and N-S offset fields in the deformation-free regions, both before and after error correction. The average of these two values was used to characterize the uncertainty of the offset field results. The uncertainties of the GNSS stations were obtained from the literature [42] and are listed in Table 6. The results listed in Table 7 show that in the raw E-W displacement field, the differences between the SPC deformation values and GNSS observations at TOWG_49 and SRT reach nearly 2 m. Six GNSS stations showing the SPC deformation values differ at about 1 m from GNSS observations. The magnitude of these differences was approximately one-fifth of the deformation caused by the earthquake. In the raw N-S displacement field, the difference between the SPC deformation value and the GNSS observations at P594_G49 was approximately 1.85 m. Three GNSS stations show SPC deformation values differing about 1 m from the GNSS observations. After error correction, the deformation results showed a remarkable 91.7% improvement in accuracy in the E-W direction and 88.9% improvement in accuracy in the N-S direction. The corrected deformation results exhibited good consistency in magnitude and direction with that of GNSS (Figure 7), with an RMSE of 12 cm in the E-W direction and 11 cm in the N-S direction.

## 5. Discussion

### 5.1. Comparison with Existing Method and Identified Limitations

Constellation satellites, leveraging their multi-satellite imaging capabilities, offer high-frequency coverage of the earth’s surface, providing redundancy and reliability compared to single-satellite systems. Even if one satellite malfunctions, others can continue working, ensuring the continuity and stability of data acquisition. Table 8 presents some common satellite constellations and their parameters. With similar spatial resolutions, RapidEye has a larger swath width compared to PlanetScope. SkySat and WorldView have sub-meter spatial resolution, but they have smaller imaging swath widths. Therefore, for large-scale disaster identification, it is necessary to consider the stitching of multi-scene images acquired at different times and under different conditions. Sentinel-2 holds an absolute advantage in imaging swath width, but its temporal resolution is relatively lower. In terms of swath width and spatial-temporal resolution, RapidEye imagery is a practical choice.

The attitude jitter distortion in the offset fields obtained from RapidEye images exhibits a banded distribution. The traditional mean elimination method can effectively correct this error, but it has limitations when the attitude jitter distortion demonstrates a linear trend. The polynomial fitting method proposed in this study can accommodate errors exhibiting either constant characteristics or linear trends. As shown in Figure 3, when n = 1000, the error displays a significant linear trend in the E-W offset field. The region (n = 500~1500) was selected to validate the effectiveness of the proposed method. After removing the satellite attitude angle error using the traditional mean elimination method, the standard deviation was 1.153 m. By applying the proposed method, the standard deviation was reduced to 1.130 m.

Furthermore, this study recognizes several limitations: (1) The SNR represents the noise level of each pixel. To enhance the accuracy of offset field analysis, we implemented a single SNR threshold to exclude pixels with low signal-to-noise ratios across the offset field. However, factors such as terrain characteristics and surface deformation also impact the SNR values. A uniform threshold for pixel exclusion may result in the loss of some deformation information. Thus, future research could set different SNR thresholds based on the surface characteristics of various regions within the offset field. For instance, in the areas of deformation, appropriately lowering the SNR threshold may facilitate the capture of more comprehensive offset field information. (2) Our study on the correction of attitude angle distortion addressed how varying pixel qualities might affect correction effectiveness. Our research lacks a discussion on whether weighting pixels based on the SNR of each pixel could enhance the effectiveness of attitude angle error correction. (3) The selected experimental area is relatively flat, neglecting the impact of terrain artifacts on the accuracy of the offset field in complex terrain environments. This error can be corrected using the method of constrained spatial baseline and radiation intensity described in [17].

### 5.2. Comparison with Current Research on the 2019 Ridgecrest Earthquake Sequences

The study employs the Ridgecrest earthquake as a case study to demonstrate the large-scale surface deformation monitoring capabilities of RapidEye. The displacement field calculated by SPC accurately captured the 2D offset field of the earthquake. The obtained offset field shows good consistency with GNSS observations in terms of deformation magnitude and direction. In addition, the maximum deformation magnitude of the earthquake obtained in this study matches the magnitude reported in [40,41]. The accuracy of SPC deformation monitoring is closely related to spatial resolution, typically ranging from 1/20 to 1/10 for the spatial resolution of optical images. The maximum horizontal deformation magnitude of the earthquake obtained in [40,41] ranges from 4 to 5 m, exhibiting a deviation of 1 to 2 m from our findings. This difference may be attributed to the higher spatial resolution of the images used by them, such as PlanetScope and WorldView, as well as the fusion of rich and multi-source satellite images. This fusion enhances the accuracy of the deformation calculation by recognizing the mutual supplementation and constraints among these images.

**Figure 8:**
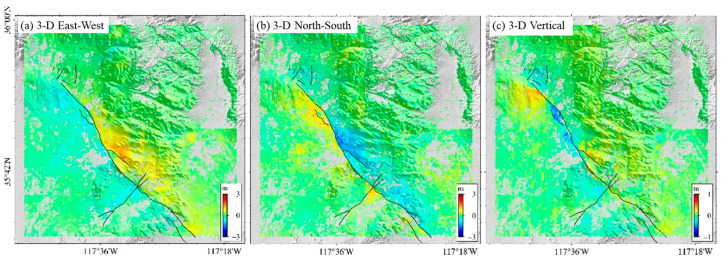
The 3D offset field of the 2019 Ridgecrest earthquake sequence.

This research performs a deformation characteristic analysis of the 2019 Ridgecrest earthquake utilizing just the 2D displacement field derived from RapidEye images without considering deformation in the vertical direction. SAR satellites, due to their unique imaging principles, have a higher sensitivity to vertical surface deformation and are important sources of three-dimensional surface deformation information. By contrast, SPC, which is based on single optical imagery, can only retrieve 2D surface deformation information. To avoid losing crucial vertical deformation information of the earthquake, the 3D offset field was created by combining data from RapidEye and Sentinel-1 before and after the event with the least square method [43] (Figure 8). As shown in Figure 8, the displacement component of fault rupture primarily emerges in the horizontal dimension, with a smaller displacement in the vertical direction. The northern portion of the main fault experiences a larger vertical deformation component than its southern counterpart, with a maximum vertical deformation magnitude of 0.5 m. This result is consistent with the vertical deformation monitoring result obtained from [44].

## 6. Conclusions

In this study, we analyzed the error characteristics of the offset fields obtained from RapidEye images and proposed correction methods. We developed an attitude jitter distortion model that considered the differences among other push-broom satellites, fully accounting for the gradient trend perpendicular to the flight direction. The polynomial fitting method effectively removed the satellite attitude jitter distortions and accommodated errors with constant characteristics or linear trends. Our investigation identified decorrelation noise, orbit error, and satellite attitude jitter distortions as the primary errors. We propose corresponding correction methods for each of these errors. Additionally, we presented a comprehensive processing workflow for monitoring surface deformation using RapidEye based on the SPC technique. Following the workflow, we obtained the 2D displacement field of the 2019 California earthquake. After error correction, fault traces of the earthquakes were effectively identified, and the deformation characteristics of the earthquake were extracted from the offset fields. The 2D offset field showed good consistency with GNSS observations in terms of magnitude and direction. In the future, improvements can be made to the error correction methods of RapidEye to enhance the automation processing. Strengthening data fusion with other monitoring methods, such as GNSS and SAR images, will enable more accurate and real-time monitoring and early warning of geological disasters such as earthquakes and landslides, thereby providing greater support for disaster prevention and control.

## Figures and Tables

**Table 1:** Parameters of RapidEye.

Parameters	Value
Orbital Altitude	630 km
Number of Satellites	5
Orbit Inclination	97.9°
Revisit Period	<1 day
Swath Width	77 km
Spatial Resolution	5 m
Band 1	Blue (440–510 nm)
Band 2	Green (520–590 nm)
Band 3	Red (630–685 nm)
Band 4	Red edge (690–730 nm)
Band 5	NIR (near infrared 760–850 nm)
Imager	Multi-spectral push-broom imager
Time on Orbit	>7 years

**Table 2:** RapidEye data information.

Pair No.	Acquisition Dates of Image Pairs Master Scene–Slave Scene	Track Num.
1	21/04/2019–29/06/2019	1155 512
2		1155 513
3		1155 612
4		1155 613
5	07/09/2019–21/09/2019	1155 512
6		1155 513
7		1155 612
8		1155 613

**Table 3:** RMSE of different bands for offset fields.

Acquisition Dates of Image Pairs	Track Num.	RMSE in E-W Direction (m)					RMSE in N-S Direction (m)				
		Band 1	Band 2	Band 3	Band 4	Band 5	Band 1	Band 2	Band 3	Band 4	Band 5
21/04/2019–29/06/2019	1155 512	0.812	0.693	0.799	0.890	0.712	0.836	0.699	0.803	0.867	0.769
	1155 513	0.949	0.746	0.899	0.982	0.813	0.898	0.664	0.756	0.823	0.691
	1155 612	0.935	0.779	0.871	0.989	0.733	0.922	0.700	0.806	0.881	0.641
	1155 613	0.960	0.940	0.832	0.940	0.856	0.774	0.711	0.772	0.834	0.640
	$\bar{RMSE}$	*0.914*	*0.789*	*0.850*	*0.950*	***0.779***	*0.858*	*0.694*	*0.784*	*0.851*	***0.685***
07/09/2019–21/09/2019	1155 512	0.686	0.598	0.634	0.746	0.597	0.821	0.638	0.625	0.710	0.531
	1155 513	0.813	0.631	0.730	0.812	0.610	0.824	0.615	0.630	0.697	0.508
	1155 612	0.893	0.667	0.666	0.731	0.676	1.001	0.714	0.656	0.698	0.600
	1155 613	0.976	0.726	0.781	0.802	0.714	0.950	0.660	0.632	0.651	0.543
	$\bar{RMSE}$	*0.842*	*0.656*	*0.703*	*0.773*	***0.649***	*0.899*	*0.657*	*0.636*	*0.689*	***0.546***

Notes: Gray highlights indicate the smallest RMSE of each coverage, and the italicized and bold portions represent the smallest mean $\bar{RMSE}$.

**Table 4:** The image data used for error source analysis.

Pairs Num.	Acquisition Dates of Image Pairs	Track Num.
1	22/04/2018–23/04/2018	1155 612
2	20/04/2019–21/04/2019	1155 612
3	07/09/2019–21/09/2019	1155 612
4	23/04/2018–20/04/2019	1155 612

**Table 5:** The information of image pairs.

Pair Num.	Master Scene–Slave Scene	Track Num.
1	20/04/2019–21/09/2019	1155 512
2	29/06/2019–06/07/2019	1155 513
3	20/04/2019–21/09/2019	1155 612
4	09/03/2019–21/09/2019	1155 613

**Table 6:** The uncertainties of the GNSS and offset field.

	P594_G49 (mm)		P595_G49 (mm)		TOWG_G49 (mm)		LGWD_G49 (mm)		Offset Field before Correcting (m)	Offset Field after Correcting (m)
Uncertainties	E-W	N-S	E-W	N-S	E-W	N-S	E-W	N-S	1.03	0.76
	2.17	2.65	2.16	2.64	2.16	2.64	1.84	1.99		

Notes: The uncertainties of the CLC, SRT, and Q0072 stations were not provided in the literature [42] and are not listed in the table.

**Table 7:** Comparison of the deformation obtained by SPC and GNSS.

	GNSS Station	GNSS Observations (m)	RapidEye before Error Correction (m)	RapidEye after Error Correction (m)	$\left|∆\right|$ before Error Correction (m)	$\left|∆\right|$ after Error Correction (m)
E-W	P594_G49	0.07	−0.75	0.09	0.82	0.02
	P595_G49	0.52	−0.36	0.30	0.87	0.22
	TOWG_G49	−0.58	1.13	−0.38	1.71	0.19
	LGWD_G49	−0.16	0.81	−0.11	0.97	0.05
	Q0072	−0.11	0.75	−0.15	0.86	0.04
	SRT	−0.25	1.58	−0.16	1.83	0.09
	CLC	0.41	0.93	0.43	0.53	0.02
N-S	P594_G49	−0.12	1.72	−0.10	1.85	0.02
	P595_G49	−0.25	−0.01	−0.23	0.25	0.03
	TOWG_G49	0.04	−0.51	0.12	0.56	0.08
	LGWD_G49	0.29	−0.54	0.12	0.83	0.17
	Q0072	0.26	−0.33	0.18	0.58	0.08
	SRT	0.10	−0.92	0.14	1.03	0.04
	CLC	−1.11	−0.43	−0.90	0.68	0.22

**Table 8:** Parameters of constellation optical satellites.

Satellite	The Number of Satellites	Spatial Resolution	Revisit Period	Swath Width
RapidEye	5	5 m	1 d	77 km
PlanetScope	170	3/4 m	2 d	20 km/24.6 km
SkySat	13	0.8/1 m	>1 d	8 km
WorldView	4	0.31/0.45/1.8/1.24 m	1 d	16 km/13 km
Sentinel-2	2	10/20/60 m	5 d	290 km

## Data Availability

The RapidEye are archived at the network of the Planet (https://www.planet.com/, accessed on 13 September 2022). The coseismic GNSS observations are derived from the network of the UNAVCO Bulletin Board (https://www.unavco.org/highlights/2019/Ridgecrest.html, accessed on 30 August 2019).
